# A Survey of the Reproductive Lesions in Captive Female Non-Human Primates in Italy

**DOI:** 10.3390/vetsci12090856

**Published:** 2025-09-04

**Authors:** Valentina Galietta, Cristiano Cocumelli, Raffaella Parmigiani, Emanuela Bovi, Tiziana Palmerini, Chiara Acri, Pilar Di Cerbo, Marco Aloisi, Antonella Cersini, Claudia Eleni

**Affiliations:** 1 Centro di Referenza Nazionale per le Malattia dei Primati non Umani, Istituto Zooprofilattico Sperimentale del Lazio e della Toscana “M. Aleandri”, Via Appia Nuova 1411, 00178 Rome, RM, Italy; 2 Fondazione Bioparco, Viale del Giardino Zoologico 20, 00197 Rome, RM, Italy; 3 Animanatura Wild Sanctuary, Strada Provinciale 155 Fibbianello, 58055 Semproniano, GR, Italy; 4 UOC Virologia, Istituto Zooprofilattico Sperimentale del Lazio e della Toscana “M. Aleandri”, Via Appia Nuova 1411, 00178 Rome, RM, Italy

**Keywords:** adenocarcinoma, endometriosis, leiomyoma, *Macaca*, non-human primates, reproduction

## Abstract

Non-human primates (NHPs) are considered important models for the study of reproductive diseases, due to their anatomical and physiological similarities to humans. However, studies on spontaneous lesions of the reproductive system in NHPs housed in zoos remain limited compared to those in laboratory animals. In this study, we report a retrospective analysis of female reproductive pathologies in 103 necropsied non-human primates from Italian zoos between 2007 and 2024. Only adult, intact, non-pregnant females with macroscopically visible reproductive lesions were included. Histopathological examination revealed reproductive tract lesions in 15 individuals (14.6%), including both non-neoplastic (cystic endometrial hyperplasia, adenomyosis, endometriosis) and neoplastic (leiomyomas, cervical and ovarian adenocarcinomas, and metastatic tumors) conditions. Leiomyoma was the most common tumor, particularly in the genus *Macaca*, while rare malignant neoplasms and metastatic lesions were identified in the great apes and in the New World species. The results suggest an age-related degenerative component and highlight interspecific differences in the distribution of lesions, probably related to the reproductive physiology of the various species. These results underline the importance of systematic post-mortem surveillance to improve the management of reproductive health of these captive populations and provide comparative insights with humans.

## Simple Summary

Non-human primates (NHPs) are considered important models for studying reproductive diseases due to their similarity to humans. However, data on spontaneous reproductive lesions in zoo-housed NHPs are limited. This study presents a retrospective analysis of female reproductive pathologies in NHPs from Italian zoos, with the aim of improving knowledge of their occurrence and comparative significance. The results highlight the importance of systematic post-mortem surveillance in enhancing reproductive health monitoring in captive populations.

## 1. Introduction

Non-human primates (NHPs) are valuable models for studying human reproductive physiology and pathology owing to their anatomical and physiological similarities to humans [1,2]. The reproductive system of NHPs, especially that of Old World primates, shares key features with that of humans, including the menstrual cycle, hormonal regulation, and susceptibility to comparable gynecological diseases [2,3,4,5,6]. These features make NHPs valuable not only for biomedical research but also for conservation in zoological institutions [2]. Diseases affecting the reproductive tract of NHPs can significantly affect fertility, health, and, in the case of laboratory animals, their usefulness as research models [7,8]. Previous studies have described spontaneous reproductive lesions in various species, including endometriosis, uterine leiomyomas, ovarian cysts, and endometrial adenocarcinoma [4,9,10,11]. The prevalence of these conditions varies by species, age, and reproductive history, highlighting the importance of systematic studies of reproductive pathology in captive populations [4]. Among the most commonly reported pathologies, endometriosis has been widely documented in rhesus macaques (*Macaca mulatta*), often showing similarities to the human disease, including chronic inflammation and fibrosis [12]. Other lesions, such as uterine leiomyomas and ovarian neoplasms, have been observed in great apes, including chimpanzees (*Pan troglodytes*) and gorillas (*Gorilla gorilla*), often presenting diagnostic and therapeutic challenges in their management [13,14,15]. Although the literature on reproductive pathology in NHPs is growing [16], much of the available data is derived from experimental studies in laboratory environments [6,8,13,15,17,18]. Systematic necropsy-based evaluations of reproductive lesions in captive primates are still limited, restricting the ability to determine their actual prevalence, clinical significance, and possible management strategies [3,19]. Comprehensive post-mortem investigations can offer valuable information about these diseases, their pathogenesis, and their impact on animal health and conservation. Through the analysis of a cohort of captive NHPs undergoing necropsy, we provide a detailed macroscopic and histopathological characterization of reproductive system lesions in different species, with the aim of increasing the knowledge on their reproductive pathology.

## 2. Materials and Methods

The cases were retrieved from the database of *National Reference Center for Non-Human Primate Diseases* at the Anatomic Pathology and Histopathology Laboratory of the Istituto Zooprofilattico Sperimentale del Lazio e della Toscana **“***M. Aleandri***”**, which archives postmortem examinations performed on deceased primates submitted to investigate the cause of death. Between 2007 and 2024 a total of 103 female NHPs were necropsied, but only adult, intact females that exhibited macroscopically evident lesions in the reproductive organs during necropsy were included in the study. Animals were submitted either refrigerated or frozen, depending on the conditions of carcass storage prior to necropsy. Cases were excluded if reproductive tissues were absent, incomplete, severely affected by post-mortem autolysis, or when only physiological uterine changes related to the menstrual cycle were observed. The animals came from two zoological gardens and a national wildlife rescue center. For each individual, data on species, age, origin, and medical history were collected, where available.

When subjects presented multiple concurrent lesions within the same organ, the pathological processes were described separately to ensure a clear comparative analysis of the different conditions observed. During the necropsy procedure, tissue samples from the reproductive tract lesions were collected, fixed in 10% neutral buffered formalin, routinely processed for paraffin embedding, and stained with hematoxylin and eosin. In selected cases, Masson’s trichrome staining was performed to confirm the muscle origin of the lesion. Immunohistochemistry was performed in the case of histiocytic sarcoma in *Pongo pygmaeus* using primary antibodies including vimentin (mAb V9, 1:100, Dako, Glostrup, Denmark), pan-cytokeratin (AE1/AE3, 1:100, Santa Cruz, Dallas, USA), CD3 (pAb, 1:200, Dako, Glostrup, Denmark), ), CD79a (mAb HM57, 1:100, Santa Cruz, Dallas, USA), CD163 (mAb AM-3K, 1:75, TransGenic Inc., Kobe, Japan), CD204 (mAb SRA-E5, 1:500, TransGenic Inc., Kobe, Japan), HLA-DR (mAb TAL 1B5, 1:100, Novus Biologicals), Iba1 (pAb, 1:300, FUJIFILM Wako, Osaka, Japan), and Ki67 (mAb MIB-1, 1:100, Dako, Glostrup, Denmark ). Serial sections of unaffected orangutan lymph nodes served as internal positive controls, while omission of the primary antibody was used as a negative control. The IHC panel was carried out on the liver, which represented the major lesion; however, morphologically identical neoplastic cells were observed in the ovary and in several other organs (including pancreas and spleen), supporting the diagnosis of disseminated histiocytic sarcoma.

All slides were independently evaluated by two experienced veterinary pathologists, with specific expertise in comparative pathology of non-human primates.

A formalin-fixed, paraffin-embedded sample of the cervical adenocarcinoma was also used for polymerase chain reaction (PCR) to detect papillomavirus, using two CODHEOP PCR protocols specifically for a highly conserved 478 bp region of the L1 gene [20] and for a highly conserved 341 bp region of the E1 gene, respectively, [21].

## 3. Results

Reproductive tract macroscopic lesions were identified in 15 out of 103 animals (14.6%) (Table 1). These individuals belonged to Old World NHPs (12/15), New World NHPs (2/15), and prosimians (1/15), with an age range between 17 and 33 years. To achieve a more precise characterization of the pathological conditions, the lesions were categorized into uterine and ovarian lesions, although some individuals presented concurrent alterations in both anatomical sites. No lesions were observed in other segments of the genital tract.

### 3.1. Uterine Pathologies

The uterine pathologies were classified into non-neoplastic and neoplastic lesions. The non-neoplastic lesions included cystic endometrial hyperplasia, adenomyosis, and endometriosis, while the neoplastic lesions comprised leiomyomas, cervical carcinoma, and metastatic tumors originating from primary neoplasms in other organs.

#### 3.1.1. Non-Neoplastic Uterine Lesions

**Figure 1:**
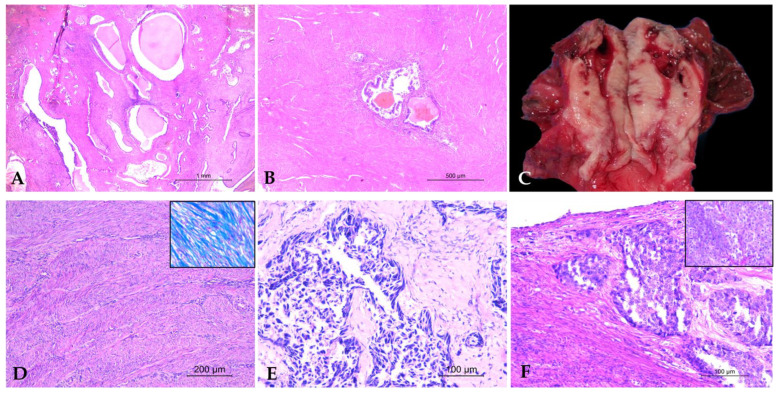
Uterine pathologies. (**A**) Endometrial glandular hyperplasia in *Ma*: cystically dilated endometrial glands with increased gland-to-stroma ratio (HE, 2.5×); (**B**) Adenomyosis in *HI*: multifocal ectopic endometrial glands within the deep myometrium (HE, 5×); (**C**) Endometriosis in *Mn*: multifocal cystic hemorrhagic areas in the uterine wall; (**D**) Leiomyoma in *Mf1*: Spindle cell proliferation arranged in interwoven bundles (HE, 10×. Inset: Masson’s trichrome staining highlights smooth muscle fibers in red); (**E**) Adenocarcinoma of the cervix in *Sa2:* Proliferation of neoplastic glandular structures in fibrovascular stroma (HE, 20×); (**F**) Metastasis of solid mammary carcinoma in *Lc*: dense sheets of neoplastic epithelial cells within the myometrium and uterine serosa (HE, 20×). Inset:primary solid mammary carcinoma.

Cystic endometrial hyperplasia was diagnosed in three individuals: *Macaca assamensis* (*Ma*), *Hylobates lar* (*HI*), and *Sapajus apella* (*Sa*). Macroscopically, the endometrium appeared thickened with multiple cystic formations of varying sizes and a brownish discoloration, which were visible on the endometrial mucosa. Histopathological examination revealed a marked hyperplasia of endometrial glands, characterized by irregularly shaped, branched, and frequently dilated structures forming cysts. The gland-to-stroma ratio was increased, and the glandular epithelium was composed of simple columnar or pseudostratified epithelium (Figure 1A). In some cases, the endometrial glands showed infiltration of neutrophils and macrophages. No significant cellular atypia was observed.

Adenomyosis was identified in three individuals: *Macaca assamensis* (*Ma*), *Hylobates lar (HI*) and *Sapajus apella* (*Sa*). Macroscopically, the myometrium appeared thickened and exhibited a denser consistency compared to normal uterine tissue. Histological examination revealed the multifocal presence of ectopic islands of endometrial glands embedded within the deep myometrium (Figure 1B). The glandular structures appeared well-organized and lacked cellular atypia, while the surrounding smooth muscle tissue exhibited hyperplasia.

A severe case of endometriosis was identified in a *Macaca nemestrina* (*Mn*) specimen. Macroscopic examination revealed a large multilobulated mass measuring 8–10 cm in the hypogastric region, with extensive involvement of the urogenital system. Adhesions were observed between the uterus, bladder, and right ureter, along with the presence of hemorrhagic cavities in the periuterine tissues. Multifocally, the uterine wall exhibited irregular cystic areas filled with blood, and a 4 cm red-brown cyst with a thin wall and hemorrhagic content was present in the left apical portion of the uterus (Figure 1C). Histopathological analysis revealed large bands of smooth muscle tissue interspersed with fibrous connective tissue in the myometrium. Multifocally, ectopic islands of hyperplastic endometrial glands and ectopic endometrial stroma were identified, disrupting and replacing smooth muscle bundles. Hemorrhagic foci and hemosiderin-laden macrophages were observed within the myometrial tissue, while the uterine lumen contained erythrocytes, homogeneous eosinophilic material, and neutrophils.

#### 3.1.2. Neoplastic Uterine Lesions

Leiomyoma was the most frequently diagnosed uterine neoplasm, identified in multiple seven individuals, including *Macaca fascicularis* (*Mfa1*), *Macaca mulatta* (*Mm*), *Macaca fuscata* (*Mf1, Mf2, Mf3*), *Pan troglodytes* (*Pt*), and *Pongo pygmaeus* (*Pp1*). Macroscopic examination revealed masses ranging in size from 2 to 7 cm, with a firm consistency, a lardaceous appearance, and a white-gray coloration. Histologically, the neoplasm consisted of a proliferation of spindle-shaped cells arranged in interwoven bundles, with oval to fusiform, “cigar-shaped” nuclei, smooth margins, and low mitotic activity (Figure 1D). Masson’s trichrome staining confirmed the muscle origin of the neoplasm (Figure 1E).

Among the neoplastic pathologies, one case of cervical adenocarcinoma was identified in a *Sapajus apella* (*Sa*) specimen. Macroscopic examination showed a 3 × 6 cm mass with a lardaceous appearance and extensive areas of necrosis and suppuration, located in the pelvic and perigenital region, originating from the cervix. Histopathological analysis revealed a proliferation of neoplastic glandular structures embedded in abundant fibrovascular stroma. The neoplastic cells, displaying a polygonal to cylindrical shape, had moderate basophilic cytoplasm, indistinct margins, and round to oval nuclei with prominent nucleoli. The mitotic count was high ranged from 1 to 2 mitoses per HPF (10–20 mitoses/2.37 mm^2^), and moderate pleomorphism was observed. No neoplastic emboli were observed. Multifocally, areas of degeneration and central necrosis were present, associated with a dense infiltrate of degenerated neutrophils. Additionally, foci of dystrophic calcification were observed. Molecular tests for papillomavirus detection were negative for both the L1 and the E1 target.

Additionally, in the uterine wall of a *Lemur catta* (*Lc*) diagnosed with solid mammary carcinoma, aggregates of neoplastic cells were observed within the myometrium and uterine serosa, consistent with metastases from the primary tumor (Figure 1F).

### 3.2. Ovarian Pathologies

#### 3.2.1. Non-Neoplastic Ovarian Lesions

**Figure 2:**
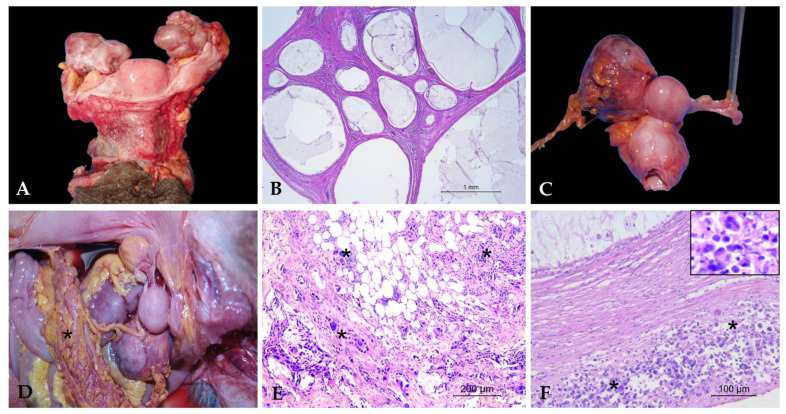
Ovarian pathologies. (**A**) Multiple follicular cysts in *Pp 1*: ovaries macroscopically altered by multiple large cysts filled with yellow-orange gelatinous material. (**B**) Multiple follicular cysts in *Pp*: cysts lined by simple cuboidal epithelium and filled with amorphous, weakly eosinophilic material (HE, 2.5×). (**C**) Ovarian adenocarcinoma in *Mfa2:* enlarged ovary with firm, lardaceous appearance and cystic areas containing brown granular material. (**D**) Ovarian carcinomatosis in *Mfa2:* numerous whitish nodules disseminated on the peritoneal surface (asterisk). (**E**) Ovarian carcinomatosis in *Mfa2:* infiltration of neoplastic epithelial cells within the peritoneal stroma (asterisks); (HE, 10×). (**F**) Metastasis of histiocytic sarcoma in ovary in *Pp 1*: multifocal infiltration of ovarian parenchyma by neoplastic cells (asterisks) (HE, 20×). Inset: detail of multinucleated neoplastic cells.

Multiple follicular cysts were identified in two *Pongo pygmaeus* (*Pp 1* and *Pp 2*). Macroscopic examination revealed cysts ranging from 2 to 6 cm in diameter, containing yellow-orange gelatinous material mixed with purulent-like content (Figure 2A). Histologically, the cysts were lined by a thin wall composed of granulosa cells supported by a theca cell layer, enclosing a cavity filled with amorphous, weakly eosinophilic material, with no evidence of cellular atypia (Figure 2B). These features were consistent with a follicular origin and allowed us to exclude SES cysts, typically lined by a single layer of flattened to cuboidal epithelium beneath the surface epithelium, and cystic rete ovarii, usually located in the ovarian hilus and lined by flattened to transitional epithelium.

#### 3.2.2. Neoplastic Ovarian Lesions

A case of ovarian adenocarcinoma was identified in a *Macaca fascicularis* (*Mfa2*). Macroscopic examination revealed an enlarged ovary with a firm consistency, a lardaceous appearance, and cystic areas containing brown granular material (Figure 2C). Histopathological analysis showed a proliferation of neoplastic epithelial cells organized into ectatic acinar structures, often containing necrotic material. The neoplastic cells exhibited marked anisokaryosis and anisocytosis, with a moderate amount of eosinophilic cytoplasm, prominent nucleoli, and pleomorfhic nuclei. The mitotic count was high (1–2 mitoses per HPF; 10–20 mitoses/2.37 mm^2^). Similar lesions were also observed in the kidneys, liver, spleen, lungs, mesentery, and urinary bladder, leading to a diagnosis of ovarian carcinomatosis (Figure 2D).

Consistent with findings in the uterus, metastatic lesions from a primary tumor located outside the reproductive tract were also identified in the ovary. In a *Pongo pygmaeus* (*Pp 1*) specimen, an ovarian metastasis from disseminated histiocytic sarcoma was diagnosed. Histological examination revealed a diffuse infiltration of the ovarian parenchyma by large round to polygonal neoplastic cells, arranged in sheets and poorly cohesive. The cells had abundant eosinophilic cytoplasm, often vacuolated, with indistinct borders. Nuclei were large, hyperchromatic, and pleomorphic, with vesicular chromatin and one or more prominent nucleoli; mitotic figures were frequent, and occasional multinucleated cells were observed. (Figure 2E). The immunohistochemical panel was performed on the liver, which represented the main lesion, and confirmed the diagnosis by showing Iba1 positivity (both cytoplasmic and membranous) and negativity for pan-cytokeratin (AE1/AE3), CD79a, and CD3, thus excluding an epithelial, B-cell, or T-cell origin [22]. Morphologically identical neoplastic cells were observed in the ovary as well as in other affected organs, including pancreas and spleen.

## 4. Discussion

The results confirm that reproductive tract disorders can occur in captive NHPs, even in the absence of clinical signs during life, as also highlighted in previous surveys on zoo-housed and research colony animals [2,8]. Although the overall prevalence is not high (14.6%), this finding is particularly relevant in the context of animals housed in zoological and rescue centers, where reproductive health monitoring is often limited to behavioral observations or occasional ultrasonographic test.

Non-neoplastic uterine lesions were identified in 6 of 15 subjects (40%), with cystic endometrial hyperplasia and adenomyosis representing the predominant findings. The occurrence of non-neoplastic lesions (40% of our cases) is consistent with previous observations in NHPs kept in controlled environments, where cystic endometrial hyperplasia and adenomyosis were among the most frequently detected uterine conditions [4,8]. A relevant finding is the relatively low prevalence of endometriosis, detected in only one macaque in our cohort. By contrast, previous studies on captive macaques and baboons reported a prevalence ranging from 25% to 66% in older females [2,11,23].

This discrepancy may not reflect a true variability in the distribution of the disease among the population under study, but can rather be attributable to limitations in the macroscopic detection of the pathology. Early or moderate-stage endometriosis can be difficult to identify during necropsy [24], especially in the absence of extensive lesions, adhesions, or grossly visible hemorrhagic cysts. Cooper et al. (2007) [24] reported that, in addition to macroscopically evident cases, endometriosis might also occur in a subclinical form, characterized by isolated stromal foci or the presence of hemosiderin, suggesting that relying solely on gross selection of tissue samples may lead to an underestimation of the actual prevalence. Although all cases included in the study underwent histological examination, tissue sampling was performed on gross findings, leading to an underestimation of mild or localized forms of the disease.

From a clinical perspective, endometriosis in women is associated with severe pain, infertility, and significant morbidity [25,26]. In contrast, clinical signs in non-human primates are rarely documented; however, experimental models in rhesus and cynomolgus macaques have reported infertility, abdominal distension, and signs of discomfort, paralleling the human condition [8]. In zoo-housed animals, such clinical information is often unavailable, and in our case, no clinical data were recorded, making correlations between pathology and clinical presentation difficult.

The presence of adenomyosis in multiple individuals, in association with advanced age, is consistent with findings reported in great apes by Chaffee et al. (2016) [4], where this lesion was among the most common in elderly and subfertile females. These data suggest a possible association between aging and the development of endometrial and myometrial alterations, similarly to what has been observed in humans [27]. Adenomyosis is frequently associated with endometriosis, as also noted in our case [24,28], and has been observed in experimental settings following prolonged estrogen exposure, further supporting the hypothesis of a hormone-dependent origin of these lesions.

Although uterine neoplasms do not represent one of the main categories of tumors affecting NHPs, they can have a significant clinical impact, particularly in aged individuals [3,4]. In our study, leiomyoma was the most common neoplasm of the female reproductive system in this study (7/15; 46.7%). This finding is consistent with previous reports, in which leiomyomas were described as the most frequently diagnosed uterine tumors in aged NHPs [3,4,15]. Specifically, Chaffee et al. (2016) [4] reported a prevalence of 60% in female chimpanzees, while Cline et al. (2008) [3] confirmed their common occurrence in *Macaca* spp. Similarly, Videan et al. (2011) [15] highlighted that uterine leiomyomata are among the most common reproductive lesions in captive female chimpanzees, often detected during routine health checks. From a histopathological perspective, uterine leiomyomas are benign smooth muscle tumors, typically well-circumscribed and characterized by a uniform fascicular pattern [29]. Although often asymptomatic, particularly in non-cycling or senescent individuals, they may, in some cases, reach considerable size, causing distortion of the uterine cavity, endometrial alterations, or compression of adjacent organs both in humans and animals [11,18,30]. Such effects can negatively impact the reproductive function or the animal welfare, especially in the context of assisted reproduction.

In human medicine, leiomyomas are among the most common tumors of the female genital tract, with an estimated prevalence exceeding 70% in women at the onset of menopause [31,32]. Non-human primates, particularly cercopithecids, share many pathogenetic, clinical, and histological features of these tumors, representing ideal comparative models for studying the pathophysiology of this tumor. The detection of uterine leiomyomas therefore underscores the importance of considering these lesions in the context of preventive medicine, especially in elderly individuals or those undergoing hormonal contraceptive treatments.

Additionally, attention should be drawn to the presence of malignant tumors, particularly metastatic forms, which despite representing a smaller proportion of cases, carry significant clinical and prognostic implications. In the present study, two cases of primary carcinoma (a cervical adenocarcinoma and an ovarian adenocarcinoma) and two cases of metastases to the genital tract from extra genital neoplasms were diagnosed, accounting for 26,7% of the individuals (4/15).

The cervical adenocarcinoma, identified in a *Sapajus apella (Sa)* individual, represents a rare tumor in non-human primates but may be underdiagnosed [24]. The observed infiltrative and necrotic gross appearance, along with high mitotic activity and marked cellular pleomorphism, suggests an aggressive behavior similar to that described in human cervical carcinoma. This neoplasm has been occasionally reported in primates belonging to Old World families such as Hylobatidae and Cercopithecidae families [3,24] and may be associated with predisposing factors such as chronic inflammation or persistent hormonal changes. Studies in *Macaca mulatta* have documented the presence of papillomavirus (RhPV-1), associated with cervical dysplastic or neoplastic lesions [32,33,34], analogous to HPV-16- and -18-associated forms in humans. In our case, we did not detect a papillomavirus infection.

Even more noteworthy and rare is the case of ovarian carcinoma with multiple metastases documented in a female *Macaca fascicularis (Mfa1*). The secondary dissemination to the liver, kidneys, spleen, lungs, mesentery, and urinary bladder is consistent with a picture of peritoneal carcinomatosis, with morphological and clinical features highly similar to those of serous ovarian carcinoma in women [35]. This case highlights that epithelial ovarian tumors in NHPs can also exhibit highly invasive and systemic behavior. Spontaneous ovarian tumors in NHPs are infrequently reported in the literature compared to other reproductive tract disorders. Retrospective studies such as that by Moore et al. (2003) [36] report an overall frequency of ovarian neoplasms ranging from 5 to 10%, including granulosa cell tumors, teratomas, and exceedingly rare cases of metastatic ovarian carcinoma.

An aspect of particular interest is the presence of multiple and concurrent lesions within individual animals, suggesting a multifactorial pathogenesis potentially influenced by hormonal, inflammatory, and/or immune-related factors. In this context, the lack of previous clinical data limits a comprehensive etiopathogenetic interpretation but highlights a significant information gap that could be addressed through systematic collection and management of clinical data in zoological facilities.

Although the total number of individuals per species is limited, data analysis allows for some preliminary observations regarding interspecific differences in the distribution of reproductive pathologies. Non-neoplastic uterine lesions (such as endometrial hyperplasia and adenomyosis) and benign uterine tumors (particularly leiomyomas) were more frequently observed in Old World NHPs and, in particular, in the genus Macaca, supporting findings in the scientific literature, where these species are considered among the most predisposed to reproductive tract disorders due to their menstrual physiology, which closely resembles that of humans. In great apes (such as *Pongo pygmaeus* and *Pan troglodytes*), both benign and malignant or metastatic neoplastic lesions were detected, suggesting that in these species as well, advanced age and prolonged hormonal exposure may play a significant role in the development of reproductive disorders. In New World NHPs and prosimians, lesions were less frequent but included significant neoplastic forms, such as cervical adenocarcinoma in *Sapajus apella* and uterine metastases from mammary carcinoma in *Lemur catta*. These observations are consistent with those recently described by Pereira et al. (2025) [16], who reported the presence of malignant neoplasms in New World species (Callitrichidae and Pitheciidae families). Notably, as in our study, no leiomyomas were reported in these species by Pereira et al. (2025) [16], supporting the hypothesis of taxonomic differences in tumor prevalence across primate groups. These differences could reflect interspecific variations in reproductive physiology and pathological susceptibility and underscore the need to strengthen health surveillance, especially on some species.

Another important aspect is the advanced age of the subjects, with a mean age exceeding 20 years. This finding, consistent with the literature [4,16,37], suggests that many of these lesions may represent degenerative changes associated with reproductive senescence. In non-human primates, this process appears to share some similarities with human menopause, although significant differences in hormonal physiology exist; for example, the absence of a complete cessation of ovarian activity in many NHP species.

From a conservation medicine and One Health perspective, our results underscore the importance of systematic post-mortem surveillance, even for non-infectious diseases. Understanding the pathological dynamics of the reproductive tract can improve the health management of captive populations, optimize breeding programs, and provide valuable comparative models for human medicine. As highlighted by Moresco et al. (2021) [2], reproductive health is a key indicator of overall animal health and the sustainability of captive populations.

## 5. Conclusions

This study documented a variety of spontaneous lesions affecting the female reproductive tract in different species of captive NHPs, highlighting the frequent occurrence of both non-neoplastic and neoplastic conditions that may negatively affect general health and reproductive capacity. The findings are in line with previous international studies and strengthen the importance of systematic surveillance of reproductive health in these animals. These data not only contribute to the understanding of comparative reproductive pathology but also serve as a valuable tool to guide clinical, reproductive, and conservation management strategies in zoological centers. In conclusion, some limitations should be acknowledged, including the retrospective nature of the study, the absence of systematic clinical information, and the relatively small number of cases per species. These aspects may have contributed to an underestimation of certain lesions or to a limited assessment of interspecific differences. Nevertheless, the present work provides novel data on reproductive pathologies in zoo-housed NHPs and highlights the importance of systematic post-mortem surveillance. Future studies integrating clinical, imaging, and pathological data in a multicenter setting will help to further clarify the prevalence and biological significance of these disorders.

## Figures and Tables

**Table 1:** Spontaneous female reproductive lesions in NHPs.

Category of NHP	Family	Common Name	Scientific Name	Code	Age	Uterus	Ovary
Old World NHP	Cercopithecidae	Assam macaque	*Macaca assamensis*	*Ma*	33	Cystic endometrial hyperplasia	
						Adenomyosis	
	Cercopithecidae	Cynomolgus macaque	*Macaca fascicularis*	*Mfa 1*	17	Leiomyoma	
	Cercopithecidae	Cynomolgus macaque	*Macaca fascicularis*	*Mfa 2*	adult		Ovarian Adenocarcinoma with peritoneal carcinomatosis
	Cercopithecidae	Japanese macaque	*Macaca fuscata*	*Mfu 1*	24	Leiomyoma	
	Cercopithecidae	Japanese macaque	*Macaca fuscata*	*Mfu 2*	24	Leiomyoma	
	Cercopithecidae	Japanese macaque	*Macaca fuscata*	*Mfu 3*	29	Leiomyoma	
	Cercopithecidae	Rhesus macaque	*Macaca mulatta*	*Mm*	30	Leiomyoma	
	Cercopithecidae	Pig-tailed macaque	*Macaca nemestrina*	*Mn*	20	Endometriosis	
						Adenomyosis	
	Hylobatidae	Lar gibbon	*Hylobates Iar*	*HI*	31	Cystic endometrial hyperplasia	
						Adenomyosis	
	Hominidae	Chimpanzee	*Pan troglodytes*	*Pt*	27	Leiomyoma	
	Hominidae	Orangutan	*Pongo pygmaeus*	*Pp 1*	45	Leiomyoma	Follicular cysts
							Metastatic histiocytic sarcoma
	Hominidae	Orangutan	*Pongo pygmaeus*	*Pp 2*	37		Follicular cysts
New World NHP	Cebidae	Tufted capuchin	*Sapajus apella*	*Sa1*	19	Cystic endometrial hyperplasia	
	Cebidae	Tufted capuchin	*Sapajus apella*	*Sa2*	27	Adenocarcinoma of the cervix	
Prosimian	Lemuridae	Ring-tailed lemur	*Lemur catta*	*Lc*	18	Metastatic mammary carcinoma	

## Data Availability

The original contributions presented in this study are included in the article. Further inquiries can be directed to the corresponding author.

## References

[1] Bauer C. (2015). The baboon (*Papio* sp.) as a model for female reproduction studies. Contraception.

[2] Moresco A., Feltrer-Rambaud Y., Wolfman D., Agnew D.W. (2022). Reproductive one health in primates. Am. J. Primatol..

[3] Cline J.M., Wood C.E., Vidal J.D., Tarara R.P., Buse E., Weinbauer G.F., de Rijk E.P., van Esch E. (2008). Selected Background Findings and Interpretation of Common Lesions in the Female Reproductive System in Macaques. Toxicol. Pathol..

[4] Chaffee B.K., Beck A.P., Owston M.A., Kumar S., Baze W.B., Magden E.R., Dick E.J., Lammey M., Abee C.R. (2016). Spontaneous Reproductive Tract Lesions in Aged Captive Chimpanzees. Vet. Pathol..

[5] Didier E.S., MacLean A.G., Mohan M., Didier P.J., Lackner A.A., Kuroda M.J. (2016). Contributions of Nonhuman Primates to Research on Aging. Vet. Pathol..

[6] Kim Y.Y., KwaK J., Kang B.C., Ku S.Y. (2025). Non-human primate: The new frontier model of female reproductive engineering. Front. Bioeng. Biotechnol..

[7] Abbott D.H., Rogers J., Dumesic D.A., Levine J.E. (2019). Naturally Occurring and Experimentally Induced Rhesus Macaque Models for Polycystic Ovary Syndrome: Translational Gateways to Clinical Application. Med. Sci..

[8] Kirejczyk S., Pinelli C., Gonzalez O., Kumar S., Dick E., Gumber S. (2021). Urogenital Lesions in Nonhuman Primates at 2 National Primate Research Centers. Vet. Pathol..

[9] Green S.L., Tolwani R.J., Waggie K.S., Otto G.M. (1999). Endometriosis and a paraovarian cyst in a rhesus macaque. Vet. Radiol. Ultrasound.

[10] Wilkinson M., Walters S., Smith T., Wilkinson A. (2008). Reproductive abnormalities in aged female *Macaca fascicularis*. J. Med. Primatol..

[11] Gall A.J., Olds J.E., Wunschmann A., Selmic L.E., Rasmussen J., Lewis A.D. (2018). Lesions of the female reproductive tract in Japanese macaque (*Macaca fuscata*) from two captive colonies. J. Zoo Wildl. Med..

[12] Kondova I., Braskamp G., Heidt P.J., Collignon W., Haaksma T., de Groot N., Otting N., Doxiadis G., Westmoreland S.V., Vallender E.J. (2017). Spontaneous endometriosis in rhesus macaques: Evidence for a genetic association with specific Mamu-A1 alleles. Primate Biol..

[13] Brown S.L., Anderson D.C., Dick E.J., Guardado-Mendoza R., Garcia A.P., Hubbard G.B. (2009). Neoplasia in the chimpanzee (*Pan* spp.). J. Med. Primatol..

[14] Stringer E.M., De Voe R.S., Valea F., Toma S., Mulvaney G., Pruitt A., Troan B., Loomis M.R. (2010). Medical and surgical management of reproductive neoplasia in two western lowland gorillas (*Gorilla gorilla gorilla*). J. Med. Primatol..

[15] Videan E.N., Satterfield W.C., Buchl S., Lammey M.L. (2011). Diagnosis and prevalence of uterine leiomyomata in female chimpanzees (*Pan troglodytes*). Am. J. Primatol..

[16] Pereira A.H.B., Rocha F.C., Carvalho M.P.S., Barbosa B.E.P., Balthazar D.A., Moreira S.B., Pissinatti A., Ubiali D.G. (2025). Female Reproductive System Neoplasms in Neotropical Primates. J. Med. Primatol..

[17] Barrier B.F., Allison J., Hubbard G.B., Dick E.J., Brasky K.M., Schust D.J. (2007). Spontaneous adenomyosis in the chimpanzee (*Pan troglodytes*): A first report and review of the primate literature: Case report. Hum. Reprod..

[18] Hanley P.W., Barnhart K.F., Satterfield W.C., McArthur M.J., Buchl S.J., Baze W.B. (2012). Obstructive uropathy secondary to uterine leiomyoma in a chimpanzee (*Pan troglodytes*). Comp. Med..

[19] DiGiacomo R.F. (1977). Gynecologic pathology in the rhesus monkey (*Macaca mulatta*). II. Findings in laboratory and free-ranging monkeys. Vet. Pathol..

[20] Forslund O., Antonsson A., Nordin P., Stenquist B., Göran Hansson B. (1999). A broad range of human papillomavirus types detected with a general PCR method suitable for analysis of cutaneous tumours and normal skin. J. Gen. Virol..

[21] Nespeca G., Grest P., Rosenkrantz W.S., Ackermann M., Favrot C. (2006). Detection of novel papillomaviruslike sequences in paraffin-embedded specimens of invasive and in situ squamous cell carcinomas from cats. Am. J. Vet. Res..

[22] Galietta V., Fonti N., Cocumelli C., Raso C., Di Cerbo P., Parisi F., Bovi E., Parmigiani R., Pietrella G., Cersini A. (2024). Histiocytic Sarcoma in a Captive Hybrid Orangutan (*Pongo sp*.): Morphological and Immunohistochemical Features. Animals.

[23] Nishimoto-Kakiuchi A., Netsu S., Okabayashi S., Taniguchi K., Tanimura H., Kato A., Suzuki M., Sankai T., Konno R. (2018). Spontaneous endometriosis in cynomolgus monkeys as a clinically relevant experimental model. Hum. Reprod..

[24] Cooper T.K., Gabrielson K.L. (2007). Spontaneous lesions in the reproductive tract and mammary gland of female non-human primates. Birth Defects Res. B Dev. Reprod. Toxicol..

[25] Saunder P.T.K., Horne A.W. (2021). Endometriosis: Etiology, pathobiology, and therapeutic prospects. Cell.

[26] Crump J., Suker A., White L. (2024). Endometriosis: A review of recent evidence and guidelines. Aust. J. Gen. Pract..

[27] Schrager S., Yogendran L., Marquez C.M., Sadowski E.A. (2022). Adenomyosis: Diagnosis and Management. Am. Fam. Physician.

[28] Ami Y., Suzaki Y., Goto N. (1993). Endometriosis in cynomolgus monkeys retired from breeding. J. Vet. Med. Sci..

[29] Dolmans M.M., Petraglia F., Catherino W.H., Donnez J. (2024). Pathogenesis of uterine fibroids: Current understanding and future directions. Fertil. Steril..

[30] Stewart E.A., Cookson C.L., Gandolfo R.A., Schulze-Rath R. (2017). Epidemiology of uterine fibroids: A systematic review. BJOG Int. J. Obstet. Gynaecol..

[31] Dagur G., Suh Y., Warren K., Singh N., Fitzgerald J., Khan S.A. (2016). Urological complications of uterine leiomyoma: A review of literature. Int. Urol. Nephrol..

[32] Giuliani E., As-Sanie S., Marsh E.E. (2020). Epidemiology and management of uterine fibroids. Int. J. Gynaecol. Obstet..

[33] Hertig A.T., MacKey J.J., Feeley G., Kampschmidt K. (1983). Dysplasia of the lower genital tract in the female monkey, *Macaca fascicularis*, the crab-eating macaque from Southeast Asia. Am. J. Obstet. Gynecol..

[34] Wood C.E., Borgerink H., Register T.C., Scott L., Cline J.M. (2004). Cervical and vaginal epithelial neoplasms in cynomolgus monkeys. Vet. Pathol..

[35] Deng K., Yang C., Tan Q., Song W., Lu M., Zhao W., Lou G., Li Z., Li K., Hou Y. (2018). Sites of distant metastases and overall survival in ovarian cancer: A study of 1481 patients. Gynecol. Oncol..

[36] Moore C.M., Hubbard G.B., Leland M.M., Dunn B.G., Best R.G. (2003). Spontaneous ovarian tumors in twelve baboons: A review of ovarian neoplasms in non-human primates. J. Med. Primatol..

[37] Lowenstine L.J., McManamon R., Terio K.A. (2016). Comparative Pathology of Aging Great Apes: Bonobos, Chimpanzees, Gorillas, and Orangutans. Vet. Pathol..

